# Vaginal Injection—Death

**Published:** 1874-02

**Authors:** 


*THE CLINIC—January*, 1874:

Vaginal Injection Followed by Death.—Dr. M. Queme reports a case to the Gazette des Hopitaux, December 4, 1873, in which a vaginal injection was ordered for a young, delicate girl aged sixteen years, affected with blenorrhagia. The injection penetrated the uterine cavity and the Fallopian tubes to the peritoneum, and was followed in a few days by death. On post-mortem there were signs of suppurative metritis and diffuse peritonitis. The author hereupon cites the recorded cases:

1. Case of MM. Bronardel and Martin under M. Gosselin at La Pitie: Simple examination by touch at the evening visit of a female aged forty-two, was followed by death in thirty-six hours. (Verbal communication by M. Lorain.) On autopsy both tubes were found dilated and filled with pus.

2. Aran cites a case of death after cauterization.

3. Case cited by M. Dolbeau in his course: Nelaton extracted a fibrous polypus situated near the entrance of the Fallopian tube. Autopsy revealed a purulent *tubitis* and the escape of pus into the peritoneum.

4. Dolbeau cites a case of death in a few hours by purulent *tubitis* observed at Lourcine.

5. M. Behier cauterized the cervix with nitric acid: Peritonitis and death, which Behier believed was caused by phlebitis.

6. M. Leteinturier, in a thesis submitted at Paris March 15, 1872, collected several cases in which intra-vaginal or intra-uterine injections were followed by peritoneal accidents, but in no case by death. He cites a passage from Aran, wherein this author says that he has made more than one hundred injections into the uterine cavity, and has yet to see even a partial peritonitis result.

